# Developmentally Regulated Sphingolipid Degradation in *Leishmania major*

**DOI:** 10.1371/journal.pone.0031059

**Published:** 2012-01-27

**Authors:** Ou Zhang, Wei Xu, Agiesh Balakrishna Pillai, Kai Zhang

**Affiliations:** Department of Biological Sciences, Texas Tech University, Lubbock, Texas, United States of America; University of Georgia, United States of America

## Abstract

*Leishmania* parasites alternate between extracellular promastigotes in sandflies and intracellular amastigotes in mammals. These protozoans acquire sphingolipids (SLs) through *de novo* synthesis (to produce inositol phosphorylceramide) and salvage (to obtain sphingomyelin from the host). A single ISCL (**I**nositol phospho**S**phingolipid phospholipase **C**-**L**ike) enzyme is responsible for the degradation of both inositol phosphorylceramide (the IPC hydrolase or IPCase activity) and sphingomyelin (the SMase activity). Recent studies of a *L. major* ISCL-null mutant (*iscl^−^*) indicate that SL degradation is required for promastigote survival in stationary phase, especially under acidic pH. ISCL is also essential for *L. major* proliferation in mammals. To further understand the role of ISCL in *Leishmania* growth and virulence, we introduced a sole IPCase or a sole SMase into the *iscl^−^* mutant. Results showed that restoration of IPCase only complemented the acid resistance defect in *iscl^−^* promastigotes and improved their survival in macrophages, but failed to recover virulence in mice. In contrast, a sole SMase fully restored parasite infectivity in mice but was unable to reverse the promastigote defects in *iscl^−^*. These findings suggest that SL degradation in *Leishmania* possesses separate roles in different stages: while the IPCase activity is important for promastigote survival and acid tolerance, the SMase activity is required for amastigote proliferation in mammals. Consistent with these findings, ISCL was preferentially expressed in stationary phase promastigotes and amastigotes. Together, our results indicate that SL degradation by *Leishmania* is critical for parasites to establish and sustain infection in the mammalian host.

## Introduction

Trypanosomatid protozoans of the genus *Leishmania* are vector-borne pathogens responsible for a variety of serious diseases known as leishmaniasis [1], [2]. During their life cycle, these organisms alternate between flagellated promastigotes living in the gut of sandflies and non-flagellated amastigotes residing in mammalian phagocytes. Current drugs for leishmaniasis are inadequate and no safe vaccine is available [3]. A better understanding of *Leishmania*-host interaction is needed to develop new cost-effective medications.

In many eukaryotes, sphingolipids (SLs) are important cell membrane components and their metabolites such as ceramide and sphingosine-1-phosphate control multiple processes including cell growth, differentiation and apoptosis [4], [5]. The most abundant SL in mammals is sphingomyelin (SM) and the degradation of SM is catalyzed by a group of sphingomyelinases (SMases), which sever the phosphodiester bond to generate ceramide and phosphocholine. In mammals, three classes of SMases (acid SMases, neutral SMases, and alkaline SMases) have been identified with distinct pH preferences [6], [7]. Among them, the Mg^2+^-dependent neutral SMases are the major contributors for mediating stress-induced ceramide production [8].

Different from mammals, fungal cells synthesize little or no SM; instead, the majority of SLs in fungi belong to inositol phosphorylceramide (IPC) and its glycosylated derivatives [9]. Besides fungi, IPC-based SLs are also found in plants and Trypanosomatids but not in mammals [10], [11]. In *Saccharomyces cerevisiae*, a neutral SMase homolog named ScISC1 is responsible for breaking down IPC into ceramide and phosphoinositol (the “IPCase” activity) [12]. ScISC1 also possesses neutral SMase activity even though *S. cerevisiae* cells do not synthesize SM [12]. ScISC1 was predominantly found in the endoplasmic reticulum during early growth but became associated with mitochondria during the later stages [13]. This change of localization might lead to the activation of ScISC1, which was dependent on anionic, mitochondrial phospholipids such as phosphatidylglycerol and cardiolipin [14]. Deletion of ScISC1 resulted in a slow growth phenotype and increased sensitivity to heat and oxidative-stress [15], suggesting that controlled ceramide production played a role in maintaining normal mitochondrial functions such as respiration or utilization of non-fermentable carbon sources[14]. Another study indicates that ScISC1-induced mitochondrial adaptation may also affect gene expression during the transition from anaerobic to aerobic metabolism [16]. In *Cryptococcus neoformans* (an opportunistic fungal pathogen), IPC degradation is mediated by CnISC1, a homolog of ScISC1 and mammalian neutral SMase [17]. The activity of CnISC1 was implicated in the survival of this organism in macrophages and its dissemination to the central nervous system in mice [17]. Unlike ScISC1 which hydrolyzes both inositol SLs and SM, CnISC1 only exerts IPCase activity [18].

Similar to fungi, *Leishmania* parasites do not synthesize SM but are highly abundant in IPC [19], [20]. Nonetheless, they possess a potent SMase activity [21], suggesting that these parasites may metabolize SM from the mammalian host. We recently identified an inositol phosphosphingolipid phospholipase C-like (ISCL) protein as the sole homolog of mammalian neutral SMase and fungal ISC1 in *L. major* [21]. Deletion of ISCL led to a complete loss of SMase and IPCase activity, suggesting this enzyme was required for the turnover of both parasite- and host-derived SLs [21]. In culture, ISCL-null mutants (referred to as *iscl^−^*) had a normal doubling time during the log phase but were more round in shape and less healthy compared to wild type (WT) parasites during the stationary phase [21]. Importantly, *iscl^−^* parasites failed to proliferate or cause pathology in either immunocompetent or immunodeficient mice [22]. This virulence defect could be fully rescued when *iscl^−^* mutants were complemented with either ScISC1 or mammalian neutral SMases, which, like ISCL, could degrade both SM and IPC [21]. However, a pure IPCase such as CnISC1 failed to restore the virulence of *iscl^−^* in mice, suggesting that the degradation of host-derived SM, not endogenous IPC, was required for *Leishmania* survival in the mammalian host [21]. The neutral SMase activity (mediated by an ISCL ortholog) is also found to be essential for the survival of *Trypanosoma brucei* (a Trypanosomatid pathogen related to *Leishmania* species) during the bloodstream stage and involved in the trafficking of variant surface glycoprotein [23].

*Iscl^−^* parasites exhibited very poor viability when they were cultured in a pH 5.0 medium (which mimicked the condition in mammalian phagolysosomes) to stationary phase [22]. This result suggests that SL degradation is important for conferring acid resistance [22]. The hypersensitivity of *iscl^−^* to acidic pH is responsible for its poor survival in murine macrophages [22], the definitive host cells for *Leishmania*. Since ISCL is both a neutral SMase and an IPCase, it is of interest to determine which activity is required for acid tolerance and whether the restoration of acid resistance is sufficient to recover the virulence of *iscl^−^* in mammals.

In this study, we analyzed the spatial and temporal expression of ISCL in *L. major*. We also probed the function of SMase and IPCase through complementation studies of *iscl^−^*. Our results indicate that SL degradation is developmentally regulated and possesses distinct roles during different stages of *Leishmania* life cycle.

## Results

### Spatial and temporal expression of ISCL in *Leishmania*

**Figure 1:**
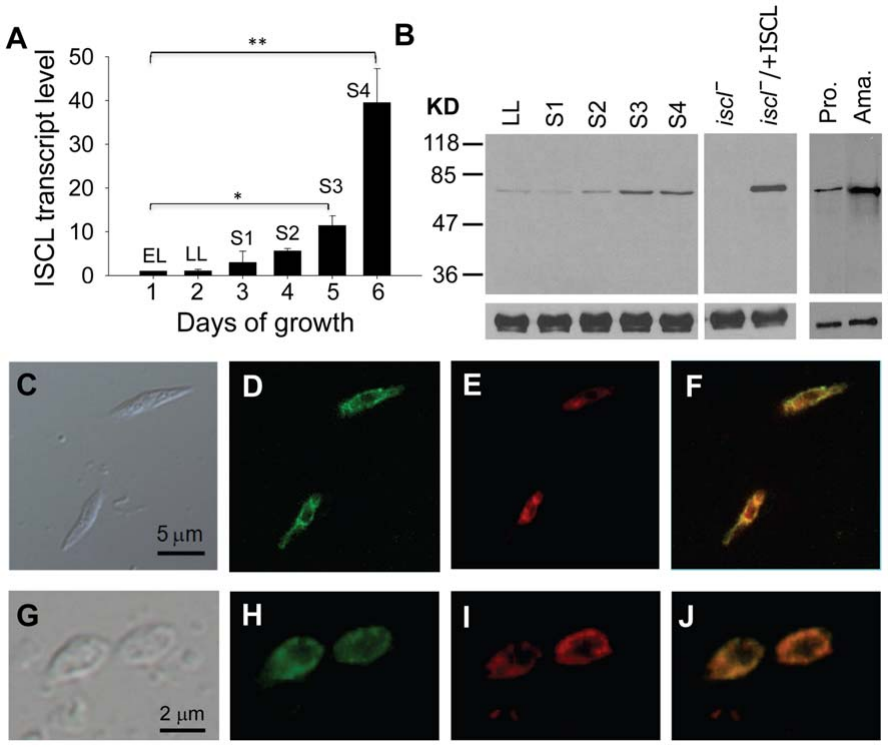
Temporal and spatial expression of ISCL in *L. major*. (**A**) Elevated ISCL transcript level in stationary phase promastigotes. *L. major* WT promastigotes were cultured in M199 medium at a starting density of 1.0×10^5^ cells/ml and total RNA was extracted daily. The relative abundance of ISCL mRNA was determined by quantative RT-PCR using the constitutively expressed rRNA45 gene as an internal control. EL: early log phase culture (1–2×10^6^ cells/ml); LL: late log phase culture (6–10×10^6^ cells/ml); S1–S4: day 1–4 stationary phase culture (2.0–3.0×10^7^ cells/ml). *: *p*<0.05, **: *p*<0.01. Error bars represent standard deviations from two independent experiments. (**B**) Increased expression of ISCL protein in stationary phase promastigotes and lesion amastigotes. Left: whole cell lysates from WT late log phase promastigotes (LL), day 1–4 stationary phase promastigotes (S1–S4), and late log phase promastigotes of *iscl^−^* and *iscl^−^/+ISCL* were subjected to immunoblot analysis using the affinity purified anti-ISCL antibody (top) or an anti-α-tubulin antibody as loading control (bottom). Right: immunoblot of cell lysates from WT day 3 stationary phase promastigotes (S3 pro.) and lesion amastigotes (ama.). Each lane contained material from 1×10^6^ cells. (**C–J**) Localization of endogenous ISCL in *L. major*. WT promastigotes (**C–F**) and amastigotes (**G–J**) were fixed and permeabilized with ethanol as described in ***Materials and Methods***. Cells were then labeled with rabbit anti-ISCL antibody, followed by staining with goat-anti-rabbit IgG-FITC (**D**, **H**) and Mitotracker Red 580 (**E**, **I**). (**F**) Overlay of **D** and **E**. (**J**) Overlay of **H** and **I**. Control cells that were labeled with secondary antibody only did not show any green fluorescence (data not shown). Experiments in **C–F** were performed three times and results from one representative set are shown here.

To determine if ISCL expression changes during *L. major* life cycle, we examined its mRNA- and protein-levels in WT parasites. First, quantitative RT-PCR analysis revealed a significant increase of *ISCL* transcript (10∼45-fold) when log phase promastigotes entered stationary phase (Fig. 1A). During the same time period, we detected an 8∼10-fold increase in the cellular abundance of ISCL protein by western-blot (Fig. 1B). The discrepancy in relative abundance between ISCL mRNA and protein may be due to posttranscriptional regulation (e.g. control of mRNA turnover and translation). ISCL protein was also highly expressed in WT amastigotes (purified from infected BALB/c mice) and *iscl^−^/+ISCL* promastigotes (the “add-back” control for *iscl^−^* in which ISCL was expressed from a high-copy vector [21]) (Fig. 1B). In *Leishmania*, late stationary phase promastigotes (containing metacyclics [24]) and amastigotes are highly infective to mammals whereas log phase promastigotes are not virulent. Therefore, the stage-dependent *ISCL* expression suggests that SL degradation may contribute to *Leishmania* infection. Also of note is that the expression profile of ISCL is opposite to that of serine palmitoyltransferase, an enzyme required for the *de novo* synthesis of SLs, which is abundant in log phase but downregulated in late stationary phase and amastigotes [20].

Previously, we examined the localization of a GFP-tagged ISCL that was overexpressed from an episomal vector and results showed that GFP-ISCL was associated with the mitochondrion in promastigotes [21]. Here we investigated the localization of endogenous ISCL in WT parasites using a rabbit anti-ISCL peptide antibody. As shown in Fig. 1C–F, the distribution of ISCL largely overlapped with the staining of mitochondrion by Mitotracker in promastigotes. A similar localization was observed in WT amastigotes isolated from *L. major*-infected mice (Fig. 1G–J). Together, these results suggest that ISCL is mainly located in the mitochondrion in *Leishmania*.

### Functional analysis of ISCL by mutagenesis

**Figure 2:**
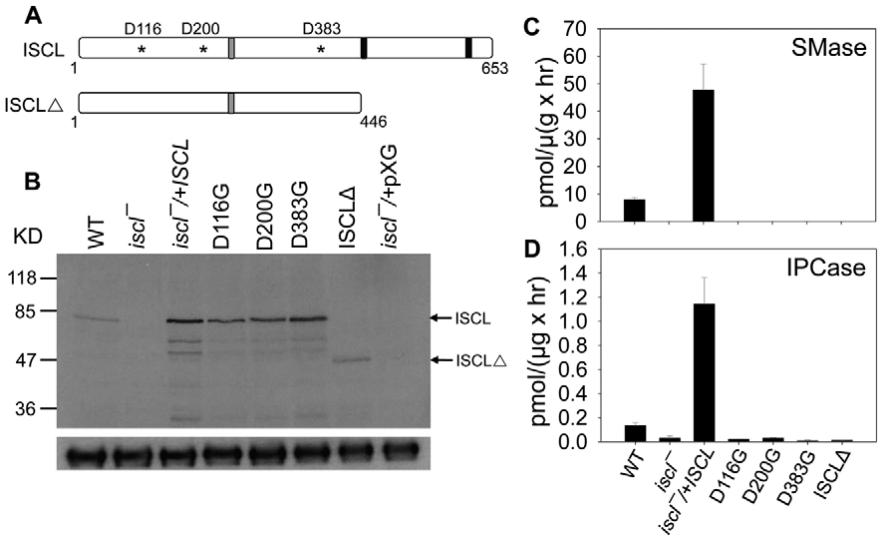
Functional analysis of ISCL by mutagenesis. (**A**) Schematic diagram of ISCL open reading frame and the mutations introduced in this study. Asterisks represent the three aspartic acids (D116, D200, and D383) that were mutated; grey bars represent the region recognized by the anti-ISCL peptide antibody; and black bars represent the transmembrane helices. (**B**) Western-blot to confirm the expression of mutated ISCL. Whole cell lysates from WT, *iscl^−^*, *iscl^−^*/+ISCL, *iscl^−^*/+ISCL D116G, *iscl^−^*/+ISCL D200G, *iscl^−^*/+ISCL D383G, *iscl^−^*/+ISCLΔ and *iscl^−^*/+pXG (empty vector) promastigotes were probed with either anti-ISCL (top) or anti-α-tubulin (bottom) antibody. Each lane contained material from 6×10^5^ cells. (**C–D**) The SMase and IPCase activities in log phase promastigotes were determined as described in ***Materials and Method***. Error bars represent standard deviations from 3 independent experiments.

Similar to its homolog in *S. cerevisiae* (ScISC1) [25], the ISCL protein contains 2 predicted transmembrane helices (AA447–466 and AA612–634) near its C-terminus (Fig. 2A and Fig. S1). Studies on ScISC1 suggest that the C-terminal domain (including those transmembrane helices) is required for anionic phospholipid binding and the tethering of N-terminal catalytic domain to mitochondrial membrane [26], [27]. For ISCL, it is not known whether these transmembrane helices are essential for its catalytic activity or its mitochondrial localization. To determine if the C-terminal domain is required for the function of ISCL, we generated a truncated ISCL (ISCLΔ) by removing the last 206 amino acid residues (AA448–653) (Figs. 2A and S1). In addition, we changed 3 Asp residues (D116, D200, and D383) in the predicted catalytic domain of ISCL to glycine through site-directed mutagenesis (Figs. 2A and S1). These Asp residues were conserved among the neutral SMase family and might be required for catalysis based on studies of the *Bacillus cereus* SMase and ScISC1 [28], [29]. In particular, D116 was located within a P-loop-like motif (AA115–121, Fig. S1) which was highly conserved in nucleotide-binding proteins and might be involved in Mg^2+^ binding [26], [28]. The change from Asp (acidic) to a neutral amino acid such as glycine could drastically affect the function of ISCL.

To test if these Asp residues and transmembrane regions were required for SMase/IPCase activity, modified forms of ISCL (D116G, D200G, D383G, and ISCLΔ) were cloned in a *Leishmania* expression vector (pXG) [30] and introduced into *iscl^−^* parasites via transfection. We then examined the SMase and IPCase activity in those *iscl^−^* transfectants (referred to as *iscl^−^*/+ISCL D116G, *iscl^−^*/+ISCL D200G, *iscl^−^*/+ISCL D383G, and *iscl* ^−^/+ ISCLΔ). As summarized in Fig. 2B–D, mutations of D116G, D200G, and D383G completely abolished the enzymatic activity of ISCL without significantly affecting protein steady state level. Removing the C-terminal domain not only ablated the function of ISCL (Fig. 2C–D), but also might reduce its stability as suggested by Western-blot (Fig. 2B). Alternatively, the lack of C-terminal region might alter the overall conformation of ISCLΔ leading to lower affinity to the anti-ISCL antibody used in the Western-blot (the peptide recognized by the anti-ISCL antibody is shown in Fig. S1). Thus, similar to ScISC1, the C-terminal domain of ISCL is essential for its catalytic activity. One possibility is that without the transmembrane regions, ISCLΔ may not be able to efficiently interact with membrane-bound substrates and phospholipids, as proposed for ScISC1 [26], [28].

**Figure 3:**
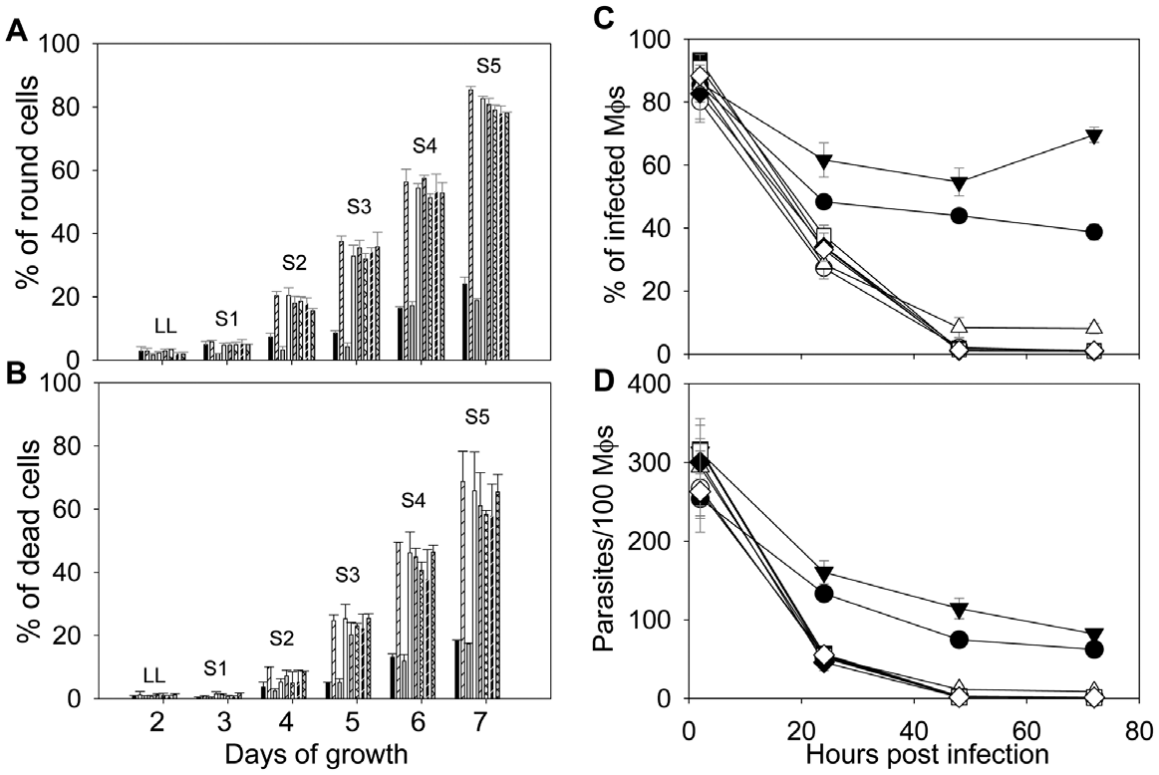
Mutated forms of ISCL failed to complement *iscl^−^* parasites. (**A–B**) Promastigotes were cultured in M199 medium and percentages of round cells (defined as those promastigotes whose long axis is shorter than twice the length of the short axis) (**A**) and dead cells (**B**) were recorded in late log (LL) and stationary phase promastigotes (S1–S5) as described [21]. Each group of bars represents cells in the following order (from left to right): WT, *iscl^−^*, *iscl^−^*/+ISCL, *iscl^−^*/+pXG, *iscl^−^*/+ISCL D116G, *iscl^−^*/+ISCL D200G, *iscl^−^*/+ISCL D383G, and *iscl^−^*/+ISCLΔ. (**C–D**) Stationary phase promastigotes (•: WT, ○: *iscl^−^*, ▾: *iscl^−^*/+ISCL, ▪: *iscl^−^*/+ISCL D116G, □: *iscl^−^*/+ISCL D200G, ⧫: *iscl^−^*/+ISCL D383G, ⋄: *iscl^−^*/+ISCLΔ) were used to infect bone-marrow derived macrophages (Mφs) from BALB/c mice at a ratio of 15 parasite per Mφ. Percentages of infected Mφs (**C**) and parasites per 100 Mφs (**D**) were determined at 2, 24, 48, and 72 hours post infection as described. As a control, WT parasites were also used to infect Mφs that were activated with 100 ng/ml of LPS and 100 ng/ml of IFN-γ (▵: WT+activated Mφs). Experiments were repeated 3 times and error bars represent standard deviations.

When introduced into *iscl^−^*, these modified ISCL proteins (D116G, D200G, D383G, and ISCLΔ) did not reverse the defects of *iscl^−^* promastigotes in culture, as the transfectants continued to show more round cells and poor viability in stationary phase (Fig. 3A–B). We also examined whether they were able to infect murine macrophages. As shown in Fig. 3C–D, without a functional ISCL, *iscl^−^* parasites could be internalized efficiently by macrophages as they were easily detected at 2 hours post infection but failed to survive after 24–28 hours. In contrast, WT and *iscl^−^*/+ ISCL parasites survived much better in macrophages (Fig. 3C–D). Therefore, the IPCase/SMase activity of ISCL is required for its function in *Leishmania*.

### The IPCase activity is required for the maintenance of cell shape and acid resistance in stationary phase promastigotes

**Figure 4:**
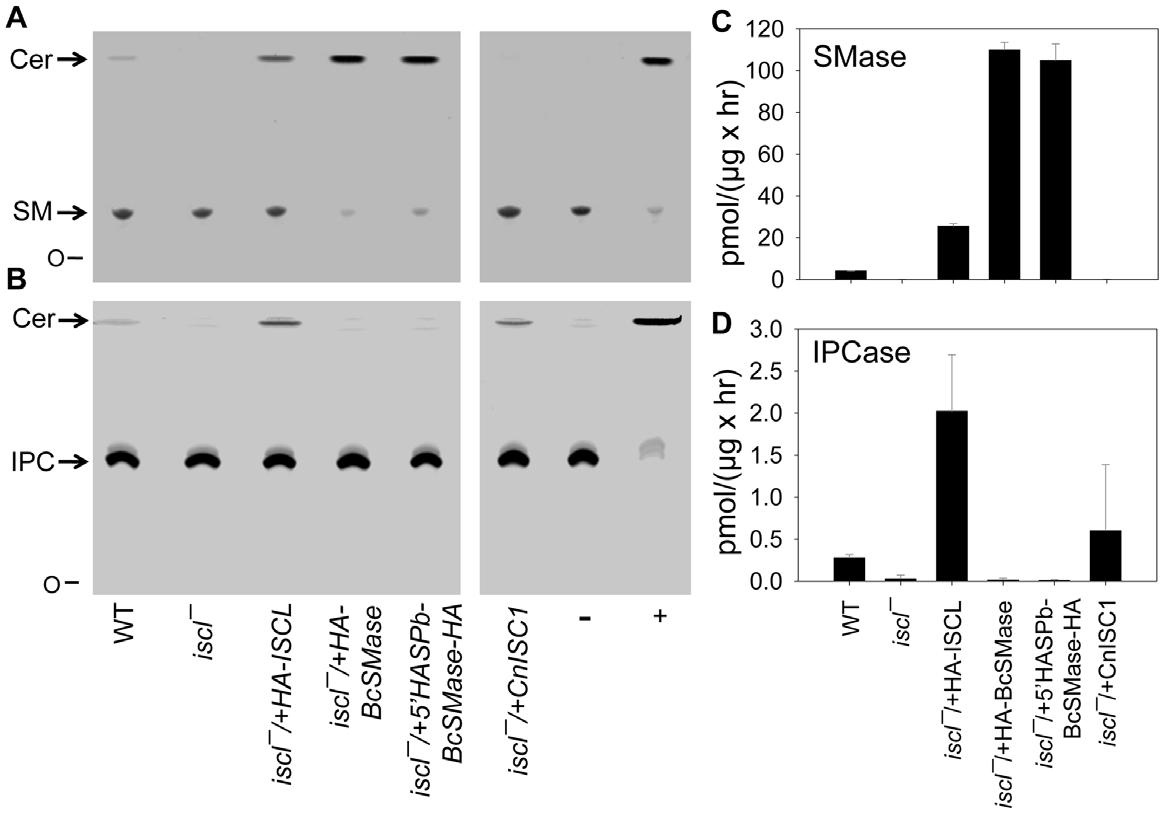
Introduction of a sole SMase or sole IPCase into *iscl^−^*. Whole cell lysates from late log phase promastigotes of WT, *iscl^−^*, *iscl^−^*/+HA-ISCL, *iscl^−^*/+HA-BcSMase, *iscl^−^*/+5′HASPb-BcSMase-HA, and *iscl^−^*/+CnISC1 parasites were incubated with NBD-labeled SM (**A** and **C**) or NBD-labeled IPC (**B** and **D**) as described in Materials and Methods. Each reaction contained ∼40 µg of total protein corresponding to 4×10^6^ cells. (**A–B**) After incubation, lipids were extracted and separated by thin layer chromatography (TLC). Ceramide (Cer), a product of SMase and IPCase, migrates faster than sphingomyelin (SM in **A**) or IPC (**B**). O: origin of migration. Positive control (+): 0.1 unit of purified *Bc*SMase (**A**) or 0.1 unit of purified *Bc*PI-PLC (**B**). Negative control (−): boiled WT lysate. (**C–D**) Activity of SMase (**C**) or IPCase (**D**) in *Leishmania* cell lysates was quantified after TLC analysis based on the amount ceramide produced and the amount of protein in each reaction. Error bars represent standard deviations from 3 independent experiments.

In culture, *iscl^−^* mutants replicated normally during log phase but died rapidly in late stationary phase, especially when they were grown in a pH 5.0 medium which mimicked the acidic condition in mammalian phagolysosomes [22]. This hypersensitivity to acid shock is responsible for the poor survival of *iscl^−^* in macrophages, as the pretreatment of macrophages with chemicals that inhibit phagolysosomal acidification significantly improved the viability of *iscl^−^* in host cells [22]. Because ISCL is both a SMase and an IPCase, we investigated which activity is required for acid resistance in stationary phase promastigotes. When the *C. neoformans* ISC1 (CnISC1) was introduced into *iscl^−^*, no SMase activity was detected (*iscl^−^*/+CnISC1 in Fig. 4A and C), although the IPCase activity was evident (*iscl^−^*/+CnISC1 in Fig. 4B and D). In contrast, when a HA-tagged *Bacillus cereus* SMase (HA-BcSMase) was introduced into *iscl^−^*, a strong SMase (*iscl^−^*/+ HA-BcSMase in Fig. 4A and C) but no IPCase activity was detected (*iscl^−^*/+ HA-BcSMase in Fig. 4B and D). As controls, WT and *iscl^−^*/+HA-ISCL parasites exhibited both SMase and IPCase activities (Fig. 4).

**Figure 5:**
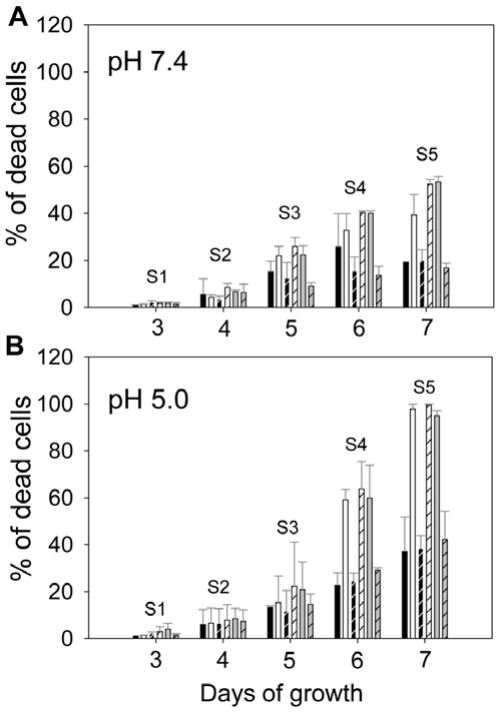
The IPCase activity was required for acid resistance in stationary phase promastigotes. Promastigotes were cultured under either neutral pH (pH 7.4, **A**) or acidic pH (pH 5.0, **B**). Cell viability in stationary phase (S1–S5) was determined by flow cytometry after staining with propidium iodide. Each group of bars represents cells in the following order (from left to right): WT, *iscl^−^*, *iscl^−^*/+HA-ISCL, *iscl^−^*/+HA-BcSMase, *iscl^−^*/+5′HASPb-BcSMase-HA, and *iscl^−^*/+CnISC1. Error bars represent standard deviations from 3 independent experiments.

**Figure 6:**
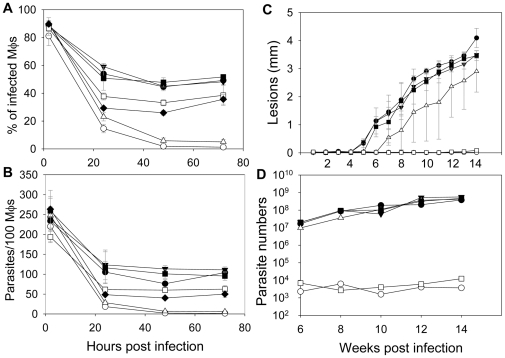
The SMase activity alone was sufficient to restore virulence in *iscl^−^*. (**A–B**) Bone marrow-derived Mφs from BALB/c mice were infected by stationary phase promastigotes (•: WT, ○: *iscl^−^*, ▾: *iscl^−^*/+HA-ISCL, ▪: *iscl^−^*/+CnISC1, □: *iscl^−^*/+HA-BcSMase, ⧫: *iscl^−^*/+5′HASPb-BcSMase-HA, ▵: WT+activated Mφs) at a ratio of 15 parasites per Mφ. Percentages of infected Mφs (**A**) and the number of parasites in 100 Mφs (**B**) were recorded. (**C–D**) BALB/c mice were infected in the footpads with day 3 stationary promastigotes (•: WT, ○: *iscl^−^*, ▾: *iscl^−^*/+HA-ISCL, ▵: *iscl^−^*/+HA-BcSMase, ▪: *iscl^−^*/+5′HASPb-BcSMase-HA, □: *iscl^−^*/+CnISC1) at 1×10^6^ parasites per mouse (5 mice per group). Lesion sizes were measured with a caliper (**C**) and parasite numbers in infected footpads were determined by the limiting dilution assay (**D**). Error bars represent standard deviations from 2 independent experiments.

Notably, after being introduced into *iscl^−^*, CnISC1 complemented the acid resistance defect (Fig. 5) and restored cell morphology in stationary phase promastigotes (Fig. S2). In addition, *iscl^−^*/+ CnISC1 parasites survived well in murine macrophages (similar to WT parasites; Fig. 6A–B). Together, these results suggest that the IPCase activity alone is sufficient to confer resistance to acid shock and help promastigotes establish infection in mammalian cells. The localization of CnISC1 in *Leishmania* is not known and it would be interesting to determine whether the mitochondrion-localization of IPCase is required for its function.

**Figure 7:**
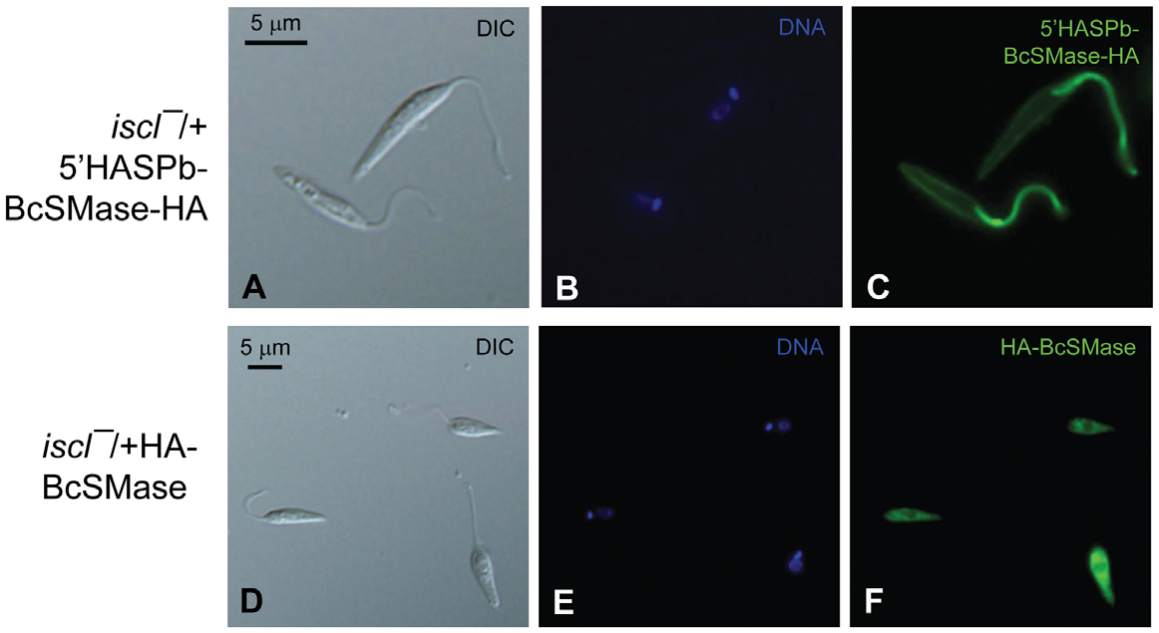
Cellular localization of HA-tagged BcSMase. Late log phase promastigotes of *iscl^−^*/+HA-BcSMase (**A–C**) or *iscl^−^*/+5′HASPb-BcSMase-HA (**D–F**) were labeled with a monoclonal anti-HA antibody followed by anti-mouse IgG-FITC. (**A**, **D**) DIC images; (**B**, **E**) Hoechst staining of DNA; (**C**, **F**) anti-HA staining. Control cells that were labeled with secondary antibody only did not show any green fluorescence (data not shown).

Next, we examined whether the SMase activity alone can complement the defects in *iscl^−^*. In addition to HA-BcSMase, we generated another SMase by fusing the first 18 amino acids of the *L. major* HASPb protein (which can target a cargo protein to cell surface including the flagellar surface) [31] to the N-terminus of BcSMase. The resulting 5′-HASPb-BcSMase-HA was similar to HA-BcSMase in that it was highly active against SM but not IPC when it was introduced into *iscl^−^* (*iscl^−^*/+5′-HASPb-BcSMase-HA in Fig. 4). As shown in Fig. 7A–C, the 5′-HASPb-BcSMase-HA fusion protein was mainly localized at the flagellar membrane although it was also visible at the cell membrane [11]. In contrast, without the HASPb leader sequence, HA-BcSMase was primarily found in the cytoplasm (Fig. 7D–F). These results allowed us to evaluate whether the localization of SMase affects its function in *Leishmania*. Importantly, despite their strong SMase activity, neither HA-BcSMase nor 5′-HASPb-BcSMase-HA restored acid resistance or cell shape in *iscl^−^* during stationary phase (Figs. 5 and S2), although they were able to somewhat improve *iscl^−^* survival in macrophages (Fig. 6A–B). Together, these studies suggest that the SMase activity alone cannot confer acid tolerance but can help *Leishmania* establish infection in macrophages.

### The SMase activity is required for amastigote proliferation in mice

Although the CnISC1 (a sole IPCase) was sufficient to complement the defects of *iscl^−^* promastigotes in culture, it could not restore their virulence as the *iscl^−^*/+ CnISC1 parasites failed to induce pathology or proliferate in BALB/c mice (Fig. 6C–D) [21]. In contrast, the BcSMase (a sole SMase) was able to restore the infectivity of *iscl^−^* in mice (Fig. 6C–D) but not cell morphology or viability in culture (Figs. 5 and S2). The localization of SMase did not appear to be crucial as both the cytoplasmic HA-BcSMase and flagellum/plasma membrane-associated 5′HASPb-BcSMase-HA allowed *iscl^−^* parasites to regain virulence and proliferate normally in mice (Figs. 6 and 7). Therefore, the SMase activity of ISCL is required for the survival and proliferation of *Leishmania* in mammals.

## Discussion

### Role of IPC degradation in *Leishmania* promastigotes

Promastigotes of *Leishmania* reside and multiply within the gut of sandflies. Six-nine days after a blood meal, the replicative procyclics differentiate into mammal-infective metacyclics in response to nutrient depletion and/or low pH [24], [32], [33]. The fact that IPCase contributes to cell shape and viability in culture suggests that ISCL may be required for the optimal development of promastigotes in sandfly. Based on its localization, ISCL may be involved in the production of ceramide in the mitochondrial membrane which could alter the permeability and/or other functions of the mitochondria. It has been postulated that promastigotes can utilize simple sugars and amino acids as energy sources under anaerobic conditions [34], [35]; however, when nutrients are depleted (e.g. during the stationary phase in culture and late stages in sandfly), parasites may be more dependent on mitochondrial metabolism to generate energy. Compromised mitochondrial function may also explain the hypersensitivity of *iscl^−^* to acidic pH, since the maintenance of cellular pH homeostasis requires energy, e.g. from ATP hydrolysis [36], [37]. Alternatively, the ISCL-induced ceramide production could confer tolerance to acid shock. In mammalian cells, SMase-generated ceramide plays important roles in stress response by activating protein kinase C-zeta, protein phosphatase 2A and cathepsin D [38], as well as by altering the structure/function of biological membranes [39]. In *Saccharomyces cerevisiae*, ScISC1 regulates cellular response to oxidative stress through the modulation of the iron level [40], [41] or a ceramide-dependent protein phosphatase [42]. In *Cryptococcus neoformans*, CnISC1 regulates the production of phytoceramide when cells are exposed to low pH, suggesting that IPC degradation may affect the plasma membrane ATPase under acidic stress [18]. Our data indicate that SMase alone (e.g. BcSMase) cannot restore cell shape and viability of *iscl^−^* promastigotes in culture (Figs. 5 and S2). This is likely due to the lack of SM in promastigotes (while IPC is plentiful in promastigotes). Further studies are needed to define the molecular mechanism by which IPCase utilizes to confer acid resistance and ensure cell survival under low nutrient conditions.

Although CnISC1 (a sole IPCase) successfully rescued *iscl^−^* in murine macrophages, it failed to restore virulence in mice (Fig. 6) [21]. These results suggest that by conferring acid tolerance, IPC degradation contributes to *Leishmania* survival and differentiation in the initial stage of infection (2–3 days), yet is insufficient for the continuous proliferation of amastigotes in mammals. One possibility is that SM is a more accessible substrate than IPC for intracellular amastigotes (see discussion below). In mouse infection, the BcSMase-complemented *iscl^−^* parasites did not show any noticeable delay in lesion formation (Fig. 6C–D), which was likely due to the relatively high dosage of infection (1×10^6^ parasites/mouse footpad). These data indicate that although the *in vitro* macrophage infection assay (usually lasting 2–5 days) is a fast and convenient way to evaluate *Leishmania* virulence, it does have limitations and cannot fully recapitulate parasite infection in animals.

### Role of SM degradation in *Leishmania* amastigotes

Our data suggest that the SMase activity of ISCL is necessary and sufficient for amastigote proliferation in mice. It is possible that SMase is needed to generate essential nutrients such as ceramide and phosphocholine. While the latter can be used towards the synthesis of phosphatidylcholine, it is not clear why ceramide production is important for amastigotes. Future studies will investigate whether the SMase-derived ceramide is needed for the generation of IPC (present in amastigotes) or other crucial SL metabolites. Alternatively, *Leishmania* amastigotes may utilize the SMase activity of ISCL to remove excess SM in the phagolysosome which is rich in recycled lipids including SM. In mammals, deficiency of lysosomal enzymes required for lipid metabolism can lead to a series of metabolic disorders known as lysosomal storage diseases (LSDs) [43]. Among these LSDs is the type A Niemann–Pick disease (a severe neurodegenerative disorder) caused by SM accumulation due to defects in the lysosomal acid SMase [44], [45]. It is not entirely clear how SM accumulation causes such a serious condition. One possibility is that the lack of SM turnover impairs the dynamics of raft-associated membrane receptors, leading to abnormality in signaling [46]. For *Leishmania* amastigotes, a lack of ISCL may lead to SM accumulation on cell surface and disrupt the membrane property, which could be detrimental to parasites.

Functional analysis of ISCL by mutagenesis revealed that three conserved ASP residues, D116, D200, and D383, were essential for both IPCase and SMase activity (Figs. 2–3). The C-terminal region of ISCL including the two predicted transmembrane helices may be required to maintain the overall conformation of the protein, as the ISCLΔ mutant is non-functional (Figs. 2–3). Alternatively, these membrane-bound motifs may be critical for the catalytic domain of ISCL to gain access to lipid-based substrates such as IPC and SM, as suggested for ScISC1 [25], [27]. Notably, transfection experiments showed that cytoplasmic- and flagellum/plasma membrane-localized SMases (HA-BcSMase and 5′HASPb-BcSMase-HA, respectively) were able to complement *iscl^−^* growth and pathogenesis in mice (Figs. 6 and 7), suggesting that the mitochondrial localization of ISCL is not required for its function during the amastigote stage. Taken together, we postulate that SL degradation possesses at least two important roles during *Leishmania* life cycle. For promastigotes, ISCL-mediated breakdown of IPC is important for parasite development during the late stages in the sandfly when nutrients become depleted and contributes to acid resistance. For amastigotes, the degradation of host SM is required for long-term parasite proliferation in mammals. Revealing the molecular mechanism by which ISCL contributes to *L. major* infection will provide new knowledge to *Leishmania*-host interaction. Future studies will also determine whether the role of SL degradation is conserved in other *Leishmania* species.

## Materials and Methods

### Ethics statement

This study was approved by the Animal Care and Use Committee at Texas Tech University (PHS Approved Animal Welfare Assurance NO. A3629-01). Mice were housed and cared for in the facility operated by the Animal Care and Resources Center at Texas Tech University. The facility was inspected monthly and animals were monitored daily by staff members. A complete range of clinical veterinary services was available on a 24-hour basis and includes consultation, diagnostic work-up and clinical care.

To minimize the pain and distress on animals without compromising the quality of research, mice were under anesthesia (through the peritoneal injection of ketamine hydrochloride/xylazine) during recurring procedures including the injection of *Leishmania* parasites into footpads, the recovery of parasites from infected mice, and the measurement of lesion size using a caliper. Usually, no more than one procedure was performed on one mouse within a week. To prevent any potential secondary infections and to reduce any potential pain/distress, mice were monitored carefully (twice a week for appearance, size, movement, and general health condition) and euthanized when the lesions became too large (>2.5 mm for footpad infection).

Proper restraining and injection techniques were employed by trained lab personnel to reduce pain and distress of animals. For the isolation of macrophages and the determination of parasite load in the infected footpads, mice were euthanized prior to the operation.

### Materials

BALB/c mice (female, 7–8 weeks old) were purchased from Charles River Laboratories International. N-[6-[(7-nitro-2-1,3-benzoxadiazol-4-yl)amino]hexanoyl]-sphingosine-1-phosphocholine (NBD-C6-sphingomyelin) and MitoTracker Red 580 were purchased from Invitrogen Corp. N-[12-[(7-nitro-2-1,3-benzoxadiazol-4-yl)amino]dodecanoyl]-sphingosine-1-phosphoinositol (NBD-C12-IPC) was custom-synthesized by Avanti Polar lipids. *Bacillus cereus* (ATCC strain 14579) was obtained from American Type Culture Collection. The Rabbit anti-ISCL peptide antibody was custom-produced by the Open Biosystems, Inc. The iScript Select cDNA synthesis kit and the iTaq SYBR Green Supermix with ROX were purchased from Bio-Rad Laboratories. All other chemicals were purchased from VWR International or Fisher Scientifics unless specified otherwise.

### Molecular constructs for HA-BcSMase, 5′HASPb-BcSMase-HA, and mutated forms of ISCL

Procedure for the generation of molecular constructs was described in the online supporting information (Supporting Information S1). Oligonucleotides used in this study were summarized in Table S1. All constructs were confirmed by restriction enzyme digestion and DNA sequencing.

### *Leishmania* culture and genetic manipulations

*L. major* LV39 clone 5 (Rho/SU/59/P) wild type (WT), *iscl^−^*, *iscl^−^*/+ISCL, and *iscl^−^*/+CnISC1 were grown in M199 medium with 10% fetal bovine serum and other supplements as described [21]. To generate *iscl^−^*/+CnISC1, *CnISC1* (accession #DQ487762) was PCR amplified from *C. neoformans* genomic DNA and cloned in the pIR1SAT vector as pIR1SAT-*CnISC1* (B107), followed by transfection into *iscl^−^*. To test if BcSMase alone could complement *iscl^−^*, pGEM-5′UTR-phleo-DST IR-HA-*BcSMase*-3′UTR (B257), pGEM-5′UTR-phleo-DST IR-5′HASPb-*BcSMase*-3′UTR (B267), or pGEM-5′UTR-phleo-DST IR-HA-*ISCL* (B240) were introduced into *iscl^−^* by electroporation [47] to generate *iscl^−^*/+HA-BcSMase, *iscl^−^*/+5′HASPb-BcSMase-HA, or *iscl^−^*/+HA-ISCL, respectively.

To test if mutated forms of ISCL were functional, pXG-ISCL D116G (B204), pXG-ISCL D200G (B208), or pXG-ISCL D383G (B211) were introduced into *iscl^−^* and referred to as *iscl^−^/+ISCL* D116G, *iscl^−^/+ISCL* D200G, *iscl^−^/+ISCL* D383G, or *iscl^−^/+ ISCLΔ*, respectively.

### Analyses of cell growth, morphology and viability

Promastigotes were inoculated in either neutral (pH 7.4) or acidic (pH 5.0) media (starting density = 1.0×10^5^ cells/ml) as previously described [22]; culture densities were monitored daily using a hemacytometer; percentages of round cells were determined by microscopy and cell viability was determined by flow cytometry after staining with propidium iodide as previously described [21]. In this study, round cells were referred to the promastigotes whose long axis is shorter than twice the length of their short axis.

### Quantitative RT-PCR

Total RNA was extracted from WT promastigotes using the Trizol reagent and the RNA quality was confirmed by formaldehyde agarose gel electrophoresis [48]. First strand cDNA was synthesized using the Bio-Rad iScript Select cDNA synthesis kit. Quantitative RT-PCR was performed using an Applied Biosystems 7500 Real-Time PCR system and the SYBR Green qPCR kit. Briefly, PCR was set up in triplicates in low-profile 96 well microtitre plates. Each 25 µl reaction mixture contained 1× Taq SYBR Green supermix with ROX, 2 µl of first strand cDNA and 100 nM of each 5′ and 3′ primer pair (P216/P217 for *ISCL* and P167/P168 for rRNA45). The PCR amplification cycle consisted of 2 min at 95°C for initial denaturation (1 cycle), followed by 10 sec at 95°C and 45 sec at 60°C (40 cycles). The relative expression level of *ISCL* was normalized to that of the endogenous *rRNA45* gene [49] using the comparative C_t_ method, also known as 2^−ΔΔ(Ct)^ method [50], [51]. C_t_ is the cycle threshold, ΔC_t_ is the difference in threshold cycles between *ISCL* and *rRNA45*, and ΔΔC_t_ equals the amount of *ISCL* normalized to *rRNA45* and relative to the mid-log phase value.

### Western-blot analysis

Promastigotes or purified amastigotes were collected and resuspended in phosphate buffered saline (PBS) at 1×10^8^ cells/ml. Cell extracts were prepared and western-blot was performed as previously described [20]. ISCL was detected with the rabbit anti-ISCL peptide antibody (1∶500) and the goat anti-rabbit IgG conjugated with HRP (1∶2000). The detection of α-tubulin with a rabbit anti-α-tubulin antibody (Sigma) (1∶8000) was used as a loading control. An enhanced chemiluminescence detection system (Perkin Elmer) was used to detect signals.

### SMase and IPCase assays

SMase and IPCase activities were assessed through *in vitro* enzymatic assays as previously described [21]. Log phase promastigotes were suspended in a lysis buffer containing 25 mM Tris pH 7.5, 0.1% Triton X100 and 1× protease inhibitor at 2.0×10^8^ cells/ml and incubated for 5 min on ice. Protein concentration was determined using a micro-BCA assay (Pierce). For neutral SMase assay, 40 µg of *Leishmania* protein (∼20 µl of lysate) was incubated in 100 µl of buffer containing 50 mM Tris pH 7.5, 5 mM MgCl_2_, 5 mM dithiothreitol, 0.1% Triton X100, 11 nmol of PtS (Avanti), 2.8 nmol of unlabeled sphingomyelin (Avanti), and 0.8 nmol of NBD C6-sphingomyelin. After incubation at room temperature for 60 min, 1 ml of chloroform, 0.5 ml of methanol, and 0.2 ml of water were added to each reaction and lipid was extracted, dried, and resuspended in 20 µl of chloroform: methanol (1∶2). Thin layer chromatography (TLC) was performed as we previously described [21] and plates were scanned with a Storm 860 phosphoimager. Results were then quantified to pmol/(µg×hour) after subtracting the value of negative control (reaction using boiled WT lysate). 0.1 unit of *Bacillus cereus* SMase (Sigma) was used as a positive control. For IPCase assay, similar experiments were performed except: 1) lysate was incubated in the absence of sphingomyelin and presence of 0.8 nmol of NBD C12-IPC; 2) TLC plates were developed in a different solvent (chloroform∶methanol∶water = 65∶24∶5); and 3) 0.1 unit of *Bacillus cereus* phosphatidylinositol phospholipase C (PI-PLC, Sigma) was used as a positive control. Because *L. major* promastigotes contain 1–2×10^8^ molecules of IPC per cell [52], [53], we took that amount into consideration while calculating the activity of IPCase.

### Macrophage infection

Bone marrow derived macrophages were obtained by inducing the BALB/c femur bone marrow with 20 ng/ml of macrophage colony-stimulating factor for 4 days, and infected with late stationary phase parasites (opsonized with 4% C57BL6 mouse serum) at a ratio of twenty parasites per one macrophage as previously described [54]. Percentages of infected macrophages and the number of parasites per 100 macrophages were determined microscopically after staining with 1 µg/ml of Hoechst 33342 as previously described [54]. As a control to show that Mφ are immunologically functional, WT parasites were used to infect Mφs that were activated with 100 ng/ml of LPS and 100 ng/ml of IFN-γ.

### Amastigote purification

Purification of lesion amastigotes was processed as described [55] with minor modifications. Footpads of WT-infected BALB/c mice were homogenized in DMEM on ice (25 ml of DMEM/footpad). Homogenates were centrifuged (5 min at 33 g at 4°C) to remove large cell debris. The supernatant was transferred into a new tube and further centrifuged at 1847 g for 10 min (4°C) to collect parasites. To lyse red blood cells, pellet was resuspended in 5 ml of 168 mM NH_4_Cl and incubated at room temperature for 10 min, and then diluted with 20 ml of DMEM. The recovered amastigotes were filtered through a 40 µm cell strainer (Fisher). After centrifugation (1847 g for 10 min at 4°C), cells were resuspended in DMEM and counted using a hemacytometer before further experiments.

### Immunofluorescence microscopy

To determine the localization of endogenous ISCL, parasites were fixed with 3.7% formaldehyde and permeablized with 100% ethanol, followed by re-hydration with phosphate buffered saline (PBS). Cells were first labeled with rabbit anti-ISCL antibody (1∶500 dilution in 0.5% BSA prepared in PBS) for 30 min. After washing with PBS (3 times), parasites were incubated with goat anti-rabbit IgG-FITC (1∶1000 dilution in 0.5% BSA) for 30 min. Cells were then washed again with PBS (3 times) and transferred to poly-L-lysine coated coverslips by centrifugation (462 g for 5 min), followed by staining with 350 nM of Mitotracker Red 580 for 30 min in darkness. Cells were then washed twice with PBS, once with 50% ethanol, and stained with 2.5 µg/ml of Hoechst 33342 for 10 min. Images were acquired using an Olympus BX51 Upright Fluorescence Microscope equipped with a digital camera.

To determine the localization of 5′HASPb-BcSMase-HA and HA-BcSMase, promastigotes of *iscl^−^*/+HA-BcSMase or *iscl^−^*/+5′HASPb-BcSMase-HA were stained with a monoclonal anti-HA antibody (Invitrogen; 1∶500) followed by anti-mouse IgG-FITC as described above.

### Mouse footpad infection

Virulence of promastigotes was assessed using susceptible BALB/c mice via footpad infection and limiting dilution assay (LDA) as previously described [56], [57]. Late stationary phase (∼3 days after reaching maximal culture density) promastigotes were centrifuged at 33 g for 5 min to remove aggregates and resuspended in DMEM. Parasites were injected into the footpads of 8-week old female BALB/c mice (5 mice per group) at 1×10^6^ cells/mouse. Lesion sizes were measured weekly using a Vernier caliper. Parasite loads in the infected footpads were assessed by LDA.

## Supporting Information

Supporting Information S1 **Procedure for the generation of molecular constructs used in this study.**
(PDF)

Figure S1 **The amino acid (AA) sequence of** ***L. major*** **ISCL.** Three aspartic acid residues (marked with asterisks) were mutated to glycine in this study (as D116G, D200G, and D383G). The boxed area (AA115–121) indicates the P-loop motif. AA241–256 (dashed underline) represente the sequence recognized by the anti-ISCL peptide antibody. The underlined C-terminal domain (AA447–653) was removed to generate ISCL▵. Two predicted transmembrane helices are highlighted in grey (AA447–466 and AA612–634).
(PDF)

Figure S2 **SMase activity alone cannot restore the morphological defect in** ***iscl^−^*** **promastigotes.** Promastigotes were inoculated in M199 medium and culture densities were monitored daily (**A**). Percentages of round cells were determined (**B**) as described in ***Materials and Methods***. S1–S6: day 1 through 6 in stationary phase.
(PDF)

Table S1 **Oligonucleotides used in this study.**
(PDF)
