# Supplementary material for: Developmentally Regulated Sphingolipid Degradation in Leishmania major
Source: PLoS One. 2012 Jan 27;7(1):e31059. doi: 10.1371/journal.pone.0031059 (PMC3267774; doi:10.1371/journal.pone.0031059)
Supplement: Supporting Information S1 — Procedure for the generation of molecular constructs used in this study. (PDF) [file pone.0031059.s001.pdf]

1. Site-specific mutagenesis of ISCL and the generation of C-terminal truncated ISCL (ISCLΔ):

Point mutations (D116G, D200G, and D383G) were introduced into the *ISCL* open reading frame (ORF) using a PCR-overlap extension method as described [1]. In general, each mutant ORF was composed of two PCR fragments, joined at the site of the mutation. First round PCRs were performed with the 5' ORF primer (P58) and the reverse primer for mutation site (P219, P221, or P223), as well as the 3' ORF primer (P59) and the forward primer for mutation (P218, P220, or P222). The two products were then served as template for the second round PCR with P58/P59. The final PCR fragments containing the desired mutations were digested with BamHI and cloned into the pXG vector [2] as pXG-ISCL D116G (B204), pXG-ISCL D200G (B208), and pXG-ISCL D383G (B211).

The C-terminal truncated *ISCL* (*ISCLΔ*) was generated by PCR from *L. major* genomic DNA using primer pair P58/P158. The PCR product (*ISCLΔ*) and cloned into the BamHI site of pXG vector as pXG-ISCLΔ (B151).

2. pGEM-5'UTR-phleo-DST IR-HA-BcSMase-3'UTR (to introduce HA-BcSMase in Leishmania):

The plasmid pGEM-phleo-DST IR-dd was a generous gift from Dr. Stephen M. Beverley (Washington University School of Medicine) [3]. To replace the dd (FKBP) domain with HA, primer pair P199/P200 (all primers used in this study are listed in Table S1) was used to amplify the DNA fragment that contains the DST IR followed by HA sequence (ATGTACCCATACGATGTTCCAGATTACGCT). The resulting product was sequenced and cloned into AflII and BamHI sites of pGEM-phleo-DST IR-dd to generate pGEM-phleo-DST IR-HA (B202). The 5'- and 3'-untranslated regions (UTRs) of *ISCL* were PCR amplified

(P235/P64 for 5'UTR and P282/P283 for 3'UTR) and cloned into pGEM-phleo-DST IR-HA to generate pGEM-5'UTR-phleo-DST IR-HA-3'UTR (B242). The open reading frame (ORF) of *BcSMase* was then amplified by PCR with primer pair P280/P250 and cloned into BamHI site of pGEM-5'UTR-phleo-DST IR-HA-3'UTR as pGEM-5'UTR-phleo-DST IR-HA-*BcSMase*-3'UTR (B257).

3. pGEM-5'UTR-phleo-DST IR-5'HASPb-BcSMase-HA-3'UTR (to introduce 5'HASPb-BcSMase-HA in Leishmania):

To generate the plasma membrane localized BcSMase, we introduced the N-terminal 18 amino acids of HASPb (5'HASPb) [4] to pGEM-5'UTR-phleo-DST IR-HA-3'UTR by replacing HA with 5'HASPb. Same cloning strategy was performed through amplifying the DST-IR with 5'HASPb (P199/P294) and the resulting DNA fragment was cloned into AflII and BamHI sites of pGEM-5'UTR-phleo-DST IR-HA-3'UTR to generate pGEM-5'UTR-phleo-DST IR-5'HASPb-3'UTR (B256). The C-terminal HA-tagged *BcSMase* ORF was digested with BamHI and cloned in pGEM-5'UTR-phleo-DST IR-5'HASPb-3'UTR as pGEM-5'UTR-phleo-DST IR-5'HASPb-BcSMase-HA-3'UTR (B267).

4. pGEM-5'UTR-phleo-DST IR-HA-ISCL 3'UTR (to introduce HA-ISCL in Leishmania):

As a control to BcSMase and CnISC1 (in Figs. 4, 6 and 7), the ORF of *ISCL* was amplified as previously described [5] and inserted between the BamHI and BglII sites of pGEM-phleo-DST IR-HA and the resulting construct was named pGEM-phleo-DST IR-HA-*ISCL* (B231). Finally, the 5'UTR of *ISCL* was cloned into SpeI site of pGEM-phleo-DST IR-HA-*ISCL* to generate pGEM-5'UTR-phleo-DST IR-HA-*ISCL* (B240).

REFERENCES FOR SUPPORTING INFORMATION

1. Maruta H, Holden J, Sizeland A, D'Abaco G (1991) The residues of Ras and Rap proteins that determine their GAP specificities. J Biol Chem 266: 11661-11668.

2. Ha DS, Schwarz JK, Turco SJ, Beverley SM (1996) Use of the green fluorescent protein as a marker in transfected Leishmania. Mol Biochem Parasitol 77: 57-64.

3. Madeira da Silva L, Owens KL, Murta SM, Beverley SM (2009) Regulated expression of the Leishmania major surface virulence factor lipophosphoglycan using conditionally destabilized fusion proteins. Proc Natl Acad Sci U S A 106: 7583-7588.

4. Denny PW, Gokool S, Russell DG, Field MC, Smith DF (2000) Acylation-dependent protein export in Leishmania. J Biol Chem 275: 11017-11025.

5. Zhang O, Wilson MC, Xu W, Hsu FF, Turk J, et al. (2009) Degradation of host sphingomyelin is essential for *Leishmania* virulence. PLoS Pathog 5(12): e1000692.
