# Supplementary material for: Developmentally Regulated Sphingolipid Degradation in Leishmania major
Source: PLoS One. 2012 Jan 27;7(1):e31059. doi: 10.1371/journal.pone.0031059 (PMC3267774; doi:10.1371/journal.pone.0031059)
Supplement: Figure S1 — The amino acid (AA) sequence of L. major ISCL. Three aspartic acid residues (marked with asterisks) were mutated to glycine in this study (as D116G, D200G, and D383G). The boxed area (AA115–121) indicates the P-loop motif. AA241–256 (dashed underline) represente the sequence recognized by the anti-ISCL peptide antibody. The underlined C-terminal domain (AA447–653) was removed to generate ISCL▵. Two predicted transmembrane helices are highlighted in grey (AA447–466 and AA612–634). (PDF) [file pone.0031059.s002.pdf]

1 MSHASTFAAGELPIRVLTFNLWGIFNSRMREARMKVFATKIEHYDVILLQEQFSVEDFDLIFQNA

66 SPVVQRTYTFRRFCSSFYGSGCAVISRYPISQAFFHTFPLQGYPEMVLHGDFFANKGAAMVRVM

130 VPVTMADGGAAKAQEVTLYTTHLVAVYEKVSQLSSWRRERYLPFRISQAISFADFIVSTSRPTDPI

196 IIGGDFNCSQRSLEVQMMLILLKRYGYDMHSVLPPPRALRDAAAAEQEREVGQRFFTYSDRNT

259 FNSMKTSYFKLLKLEADIPSQIDHMFFSRPAFALLQFADCPDVADGYPCVLQDAPNGLVVFTKNE

324 VHVPPHSAWYGSLWHQLFSGKRVPRGANATGQLAKLRCKAASSTEGQSADDAAHYYPMSDH

385 FGVAALLGMRVEKVDSTTAMICSSAGTGGTAALALTPEEARAVQTVVAFLEDYVRKLRSQAKTA

449 RYMAVFSLLLVATNIWVLRRLSAKEEARSAAVLERIYDMAAAATRDTAMVVQPGKGLESIKHGF

513 NTAKDWVNNQAHLTLHIVSKFTGGTPAPGINACDTPEGSKQPAANVADPSEPVPRTVPASTTAT

577 TAKRATTTPEGDAAARPDFRAIAEALTVRPLYASAWVSSAFNITAAVVGTVSFAIGVFQRAGNAN

642 VLEEQAHQLKKL

Figure S1. The amino acid (AA) sequence of *L. major* ISCL. Three aspartic acid residues (marked with asterisks) were mutated to glycine in this study (as D116G, D200G, and D383G). The boxed area (AA115-121) indicated the P-loop motif. AA241-256 (dashed underline) represented the sequence recognized by the anti-ISCL peptide antibody. The underlined C-terminal domain (AA447-653) was removed to generate ISCL△. Two predicted transmembrane helices were highlighted in grey (AA447-466 and AA612-634).
