# Supplementary material for: Developmentally Regulated Sphingolipid Degradation in Leishmania major
Source: PLoS One. 2012 Jan 27;7(1):e31059. doi: 10.1371/journal.pone.0031059 (PMC3267774; doi:10.1371/journal.pone.0031059)
Supplement: Figure S2 — SMase activity alone cannot restore the morphological defect in iscl− promastigotes. Promastigotes were inoculated in M199 medium and culture densities were monitored daily (A). Percentages of round cells were determined (B) as described in Materials and Methods . S1–S6: day 1 through 6 in stationary phase. (PDF) [file pone.0031059.s003.pdf]

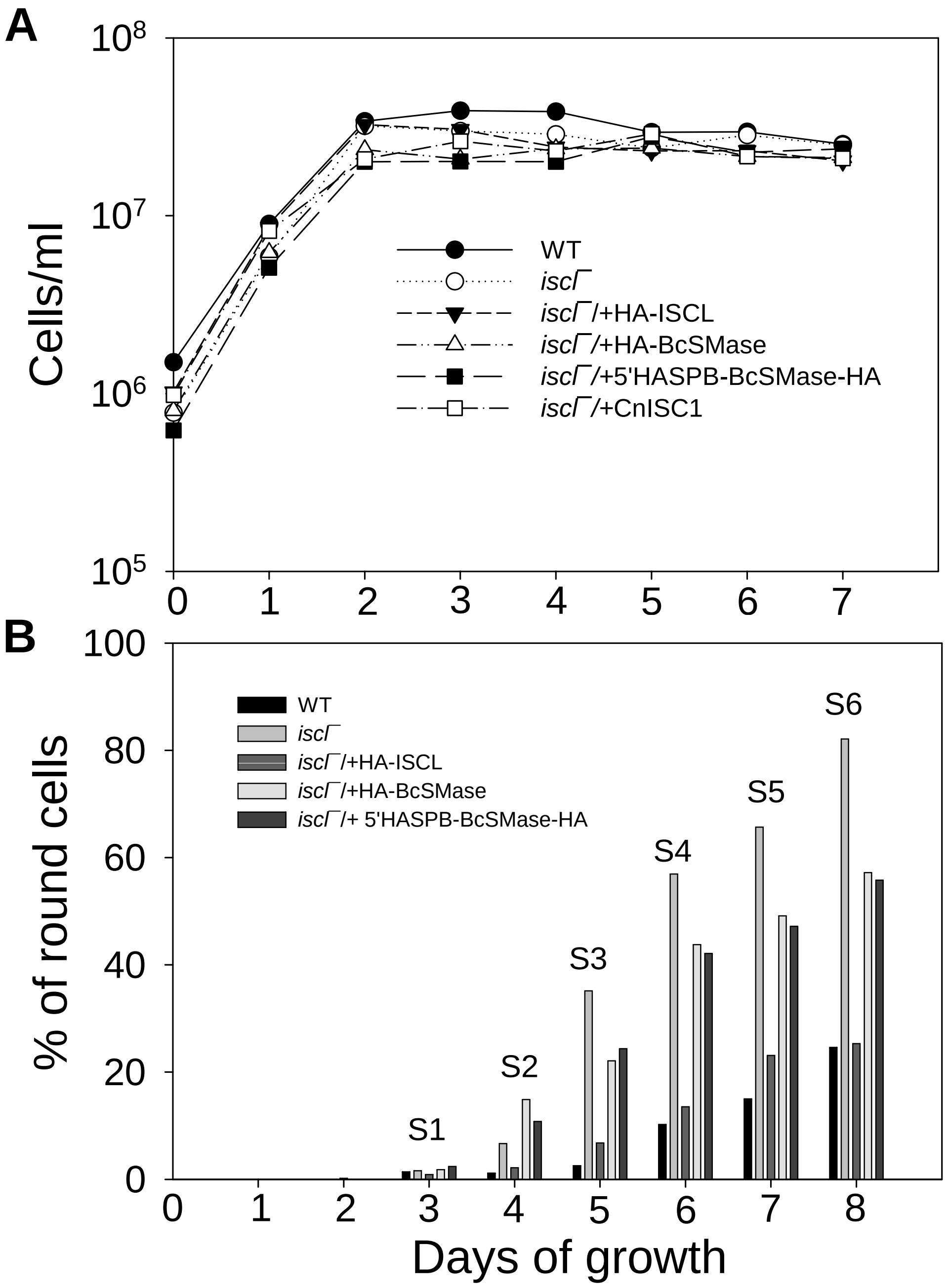

Figure S2. SMase activity alone cannot restore the morphological defect in *iscl*⁻ promastigotes. Promastigotes were inoculated in M199 medium and culture densities were monitored daily (**A**). Percentages of round cells were determined (**B**) as described in ***Materials and Methods***. S1-S6: day 1 through 6 in stationary phase.
