# Supplementary material for: Developmentally Regulated Sphingolipid Degradation in Leishmania major
Source: PLoS One. 2012 Jan 27;7(1):e31059. doi: 10.1371/journal.pone.0031059 (PMC3267774; doi:10.1371/journal.pone.0031059)
Supplement: Table S1 — Oligonucleotides used in this study. (PDF) [file pone.0031059.s004.pdf]

Table S1. List of oligonulcleotides used in this study

Primer#	Name	Purpose	Sequence
235	Forward 5'UTR (SpeI) ISCL	Synthesis of 5’UTR of ISCL	CCTCCTactagtGATACACGTGCCTCTTGCTC
64	Reverse 5’UTR ISCL (SpeI + BamHI)	Synthesis of 5’UTR of ISCL	TGCTGTGGATCCAGTTGGACTAGTGTTTTGCAGGTCTCTGTGTGTG
282	Forward 3'UTR (BglII/HindIII) ISCL	Synthesis of 3’UTR of ISCL	ATTACCAGATCTAAGCTTCAAGTCGGACAACTGG
283	Reverse 3'UTR (BglII) ISCL	Synthesis of 3’UTR of ISCL	GGTAATAGATCTCTTCTCTGTGTGCTCTTC
280	5' BcSMase ORF	Forward primer for BcSMase ORF	attactGGATCCACCATGAAAGGTAAATTGCT
281	3' BcSMase-HA	Reverse primer for BcSMase ORF with HA tag	AATCACGGATCCCTAAGCGTAATCTGGAACATCGTATGGGTACATC TTCATAGAAATAGTCGCCTC
199	Afl II DST	To amplify the DST IR (forward)	ATGAGGCCTTAAGGCAGGAAAG
200	BamHI HA-DST	Replace DST IR-dd with DST IR-HA	aatcacGGATCCAGCGTAATCTGGAACATCGTATGGGTACATGGTGGT GATCCCCCGGGG
250	3' BcSMase ORF	Reverse primer for BcSMase ORF	attactGGATCCCTACTTCATAGAAATAGTCGCCTC
294	Reverse 5'HASPb (BamHI)	Replace DST IR-dd with DST IR-5’HASPb	ATTACTGGATCCATCCGCACTTTTCTGGGGCTCCTTTGCGGAGTCCT TCGTGCAAGAGCTTCCCA
218	D116G forward	D to G point mutatation at AA116 of ISCL	GGTCCTTCACGGAGgCTTCT
219	D116G reverse	D to G point mutatation at AA116 of ISCL	AGAAGcCTCCGTGAAGGACC
220	D200G forward	D to G point mutatation at AA200 of ISCL	CATCATCGGCGGCGgCTTCA
221	D200G reverse	D to G point mutatation at AA200of ISCL	TGAAGcCGCCGCCGATGATG
222	D383G forward	D to G point mutatation at AA383of ISCL	CTACCCCATGTCGGgCCACT
223	D383G reverse	D to G point mutatation at AA383of ISCL	AGTGGcCCGACATGGGGTAG
58	5’ ISCL ORF (BamHI)	Forward primer for ISCL ORF	attactGGATCCACCATGTCGCACGCATCGACCTT
59	3’ ISCL ORF (BamHI)	Reverse primer for ISCL ORF	attactGGATCCCTACAACTTCTTCAGCT
158	3' trunc-ISCL	Reverse primer for truncated ISCL ORF	ATTACTGGATCCCTATGTCTTGGCCTGTGAGC
216	ISCL RT-Forward	Forward primer for ISCL qRT-PCR	TCAATCTCTGGGGCATCTTC
217	ISCL RT-Reverse	Reverseprimer for ISCL qRT-PCR	CTGATGGGGTAGCGTGAGAT
167	45 rRNA-Forward	Forward primer for rRNA45 qRT-PCR	CCTACCATGCCGTGTCCTTCTA
168	45 rRNA -Reverse	Reverse primer for rRNA45 qRT-PCR	AACGACCCCTGCAGCAATAC
